# Novel 14-nm Scallop-Shaped FinFETs (S-FinFETs) on Bulk-Si Substrate

**DOI:** 10.1186/s11671-015-0958-4

**Published:** 2015-06-02

**Authors:** Weijia Xu, Huaxiang Yin, Xiaolong Ma, Peizhen Hong, Miao Xu, Lingkuan Meng

**Affiliations:** Key Laboratory of Microelectronics Devices & Integrated Technology, Institute of Microelectronics, Chinese Academy of Sciences, Beijing, 100029 China

**Keywords:** FinFET, Field-effect transistors, Si nanowire, Drain-induced barrier lowering, Subthreshold swing, 85.30

## Abstract

In this study, novel p-type scallop-shaped fin field-effect transistors (S-FinFETs) are fabricated using an all-last high-k/metal gate (HKMG) process on bulk-silicon (Si) substrates for the first time. In combination with the structure advantage of conventional Si nanowires, the proposed S-FinFETs provide better electrostatic integrity in the channels than normal bulk-Si FinFETs or tri-gate devices with rectangular or trapezoidal fins. It is due to formation of quasi-surrounding gate electrodes on scalloping fins by a special Si etch process. The entire integration flow of the S-FinFETs is fully compatible with the mainstream all-last HKMG FinFET process, except for a modified fin etch process. The drain-induced barrier lowering and subthreshold swing of the fabricated p-type S-FinFETs with a 14-nm physical gate length are 62 mV/V and 75 mV/dec, respectively, which are much better than those of normal FinFETs with a similar process. With an improved short-channel-effect immunity in the channels due to structure modification, the novel structure provides one of possibilities to extend the FinFET scalability to sub-10-nm nodes with little additional process cost.

## Background

To overcome serious scaling issues, multi-gate fin field-effect transistors (FinFETs) with 3D fin-shaped channels have been extensively explored for many years as a new device platform and have been recently introduced into mass production with cutting-edge process technologies [1, 2]. Excellent short-channel-effect (SCE) immunity is achieved for FinFETs owing to the strong gate control of the double gates in the fully depleted fin channels. In addition, the fabrication technology of FinFETs is a quasi-planar process and more compatible with the conventional planar process than previous vertical double-gate devices.

To increase the scalability of a conventional SOI FinFET with double gates on the rectangular fins, tri-gate FETs, a FinFET variant with triple gates on the fins, were developed [2]. In this technology, a trapezoidal fin on a normal bulk-Si substrate is implemented to decrease process integration issues and the cost of mass production. As CMOS technology continuously scales to the 10-nm node and beyond, the nearly perfect electrostatic integrity in the fin channels due to the double or triple gates is degraded, leading to stronger SCE in the ultimate short channels [3, 4]. Some new technologies such as the skinny fin channel with a high fin height-to-width ratio and the fin channel with novel gate electrodes, such as the Π-gate or the Ω-gate, have been developed to increase the scalability of FinFETs [5, 6].

Moreover, devices with a surrounding gate, such as gate-all-round (GAA) devices or nanowire (NW) transistors, are widely recognized as the definitive solution to suppress the SCE for the strongest gate control during Si FET scaling [7, 8]. However, although GAA or NW devices have the best scalability, they suffer from the process integration challenges for large-scale CMOS production. The challenges of the surrounding gate etch and the film-gap fill beneath the NW channels prevent the implementation of state-of-the-art high-k/metal gate (HKMG) stacks and the landing pads to anchor the stacked NWs, which pose limitations on device pitch scaling for high density circuit applications [9].

In this study, to combine the scaling advantage of the surrounding gate of NW devices with the normal bulk-Si FinFET process for future mass production, a novel HKMG FinFET with a scallop-shaped fin is proposed for the first time. The structural advantages and fabrication flow are presented. The electrical characteristics and excellent SCE immunity for 27-nm and 14-nm devices are analyzed in details.

## Methods

**Fig. 1:**
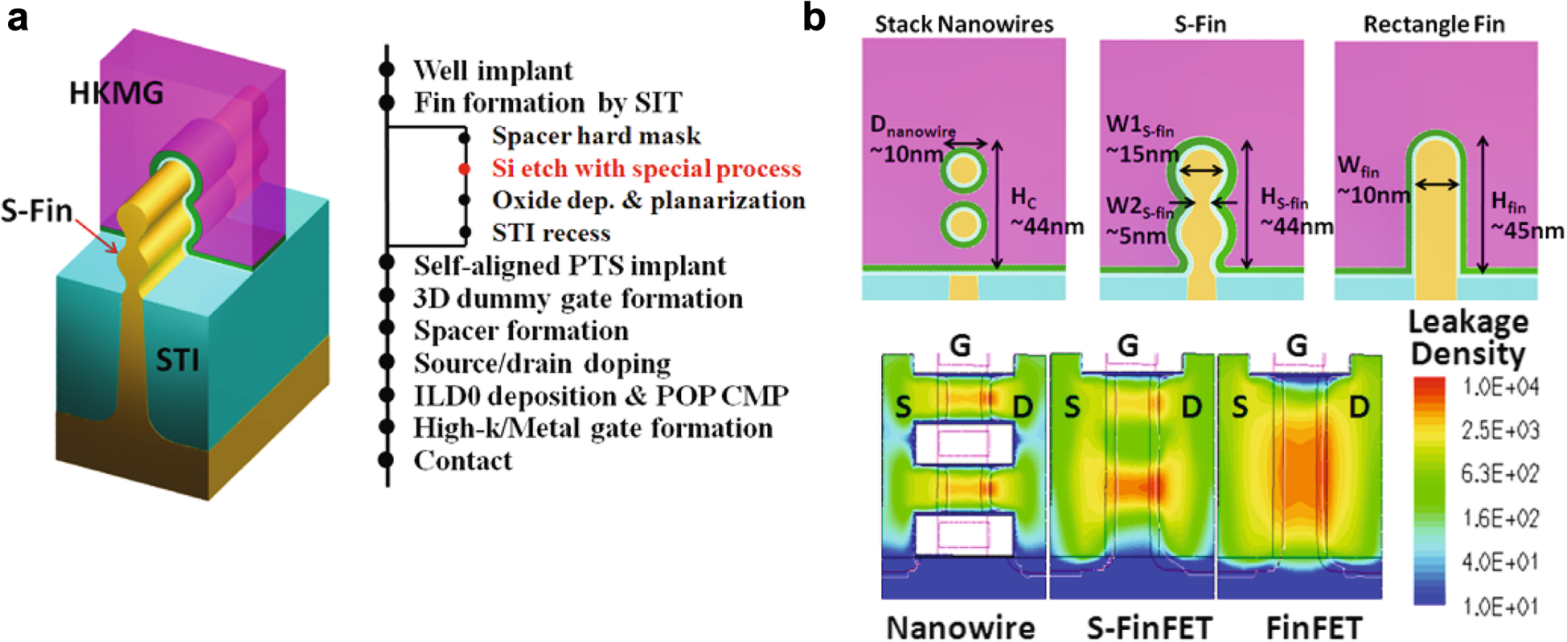
Schematic illustrations of S-FinFET design and channel control capability. **a** Design diagrams of device structure and process integration flow for S-FinFETs; **b** channel cross-sectional views and TCAD simulated channel leakage density mapping images for nanowire FET, normal FinFET, and S-FinFET

The proposed novel FinFET with a special fin structure is shown in Fig. 1a. Based on the normal FinFET structure, the sidewall profiles of the rectangular fin are modified to form continuously curved surfaces on both sides. The modified profile is similar to a scallop edge; therefore, the novel device is called a scallop-shaped FinFET (S-FinFET). The main fabrication process flow of the S-FinFETs on bulk-Si substrates is also proposed in Fig. 1a.

The entire integration flow of the S-FinFETs is fully compatible with the mainstream all-last HKMG FinFET process [2], except for a modified fin etch process.

Three different channel profiles for a normal FinFET, a stacked NW device, and an S-FinFET are shown in Fig. 1b. The S-fin has an embossed body region with a large fin width (*W1*_S-fin_) and an incurved neck region with a small fin width (*W2*_S-fin_). The stacked body channels are similar to the stacked quasi-NW channels joined together with a string. The stacked body channels with semi-surrounding gate electrodes provide stronger gate electric-field control than those of normal FinFETs or tri-gate FETs. Meanwhile, the narrow neck region has an ultrathin channel thickness, resulting in stronger gate control than conventional depletion devices with single or double gates.

In Fig. 1b, *W1*_S-fin_ and *W2*_S-fin_ of the S-FinFET are chosen as 15 and 5 nm, respectively, which are similar to the structure parameters of the fabricated S-FinFET devices presented in later paragraphs. To compare channel electrostatic integrity between different devices with a similar gate area, both the fin width (*W*_fin_) of normal vertical FinFET and the channel diameter (*D*_nanowire_) of NW device are defined as 10 nm in Fig. 1b. TCAD simulations are performed for these structures with gate length (*L*_*G*_) of 14 nm. The mapping images of the simulated leakage density (*V*_GS_ = 0 V, *V*_DS_ = 0.8 V) across different fin channels are shown in Fig. 1b. The S-FinFET demonstrates a smaller leakage distribution than normal FinFET, but is a little worse than NW device. It indicates improved channel control for the new structure over normal FinFETs.

**Fig. 2:**
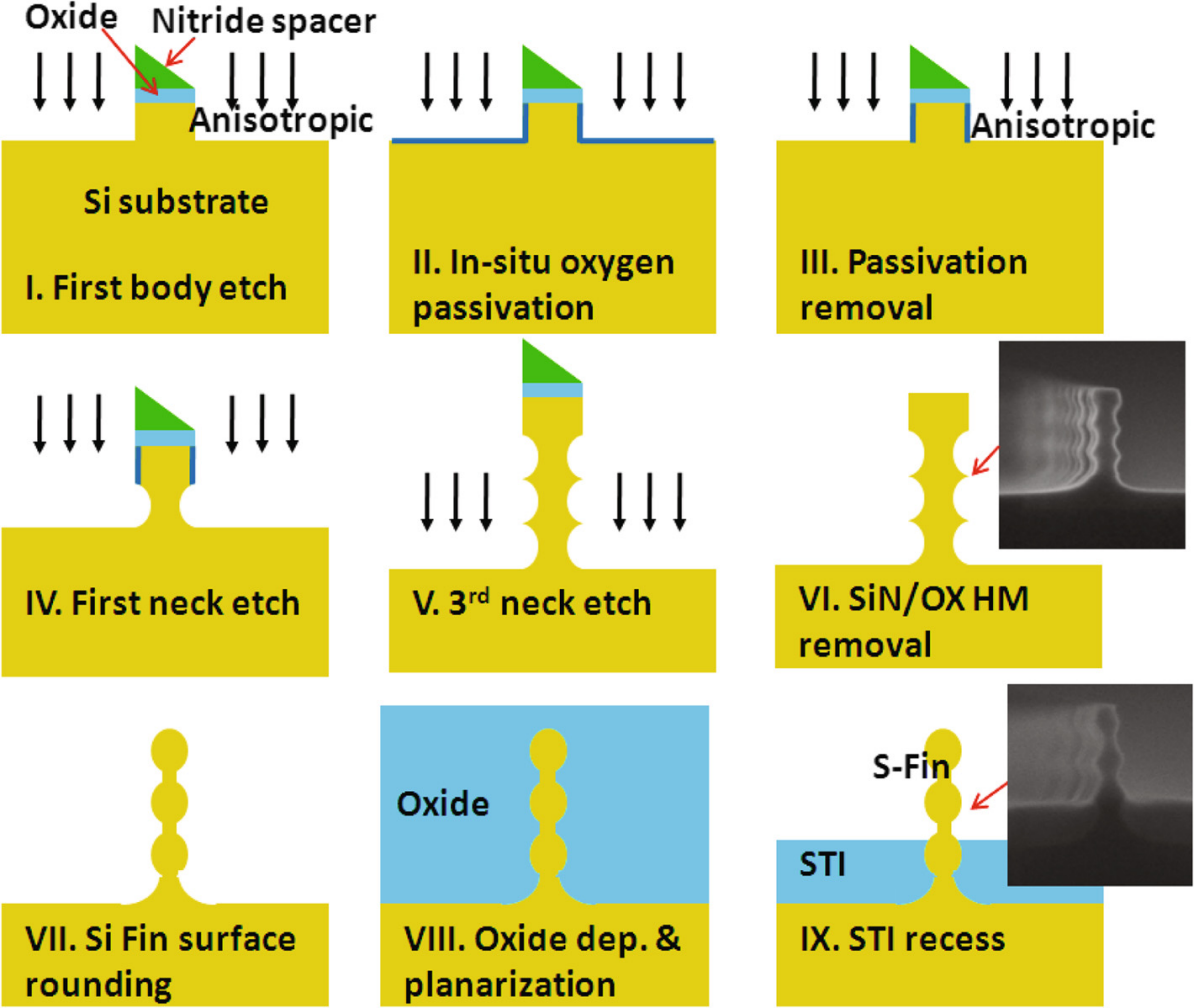
Schematic illustrations of S-fin fabrication with modified process flow. Fabrication process for forming special S-Fin with stacked fin body and fin neck channels in the traditional integration flow

The process flow for a scallop-shaped fin formation is shown in Fig. 2.

All process is carried out on our experimental fab-line. The general spacer image transfer (SIT) technique is used to create a fine 3D fin structure on the Si substrate with well doping of 1.1 × 10^17^ cm^−3^. The sacrificial core material of amorphous silicon (a-Si) is deposited on the pad oxide and patterned into multiple narrow bars by optical lithography and plasma etching. A thin conformal nitride layer is then deposited over the patterned core layer, and an anisotropic etch is subsequently performed to form a nitride/oxide spacer hard mask.

After removal of the core material, a newly developed Bosch process [10] is applied to etch Si between the spacer hard masks and to fabricate the fin array with a scallop-shaped profile. In Fig. 2, the HBr/Cl_2_ plasma is used for the initial anisotropic Si etch (step-I), following by a special O_2_/N_2_ plasma to protect the straight fin surface (step-II); thirdly, the Cl_2_ plasma is performed in next isotropic etch to form incurved fin (step-III). The chamber pressure and power are strictly controlled to precisely define the lateral etch depth. By repeating the sandwich-like etch process, the S-fin is created after a rounding and surface repairing process of a 5-nm liner oxidation (step-VII). Similar to the mainstream bulk-Si FinFET process for mass production, oxide shallow trench isolations (STI) are formed in the sequential steps (step-IX).

After S-fin formation, channel doping by punch-through stopping (PTS) implantation, dummy gate stacking, source/drain (S/D) engineering, and all-last HKMG processing are performed for the fabrication of the transistors. The conditions of PTS implantation are carefully designed for a low doping (~3 × 10^17^ cm^−3^) in the top channel. The shallow extension regions of S/D are formed with twice 3 keV BF^+^ implantations with tilted angle of 30° against to the direction of channel followed by a spike annealing. As a reference, FinFETs with conventional trapezoidal fins are fabricated with the same process, except for the special Si fin etch process.

## Results and discussion

**Fig. 3:**
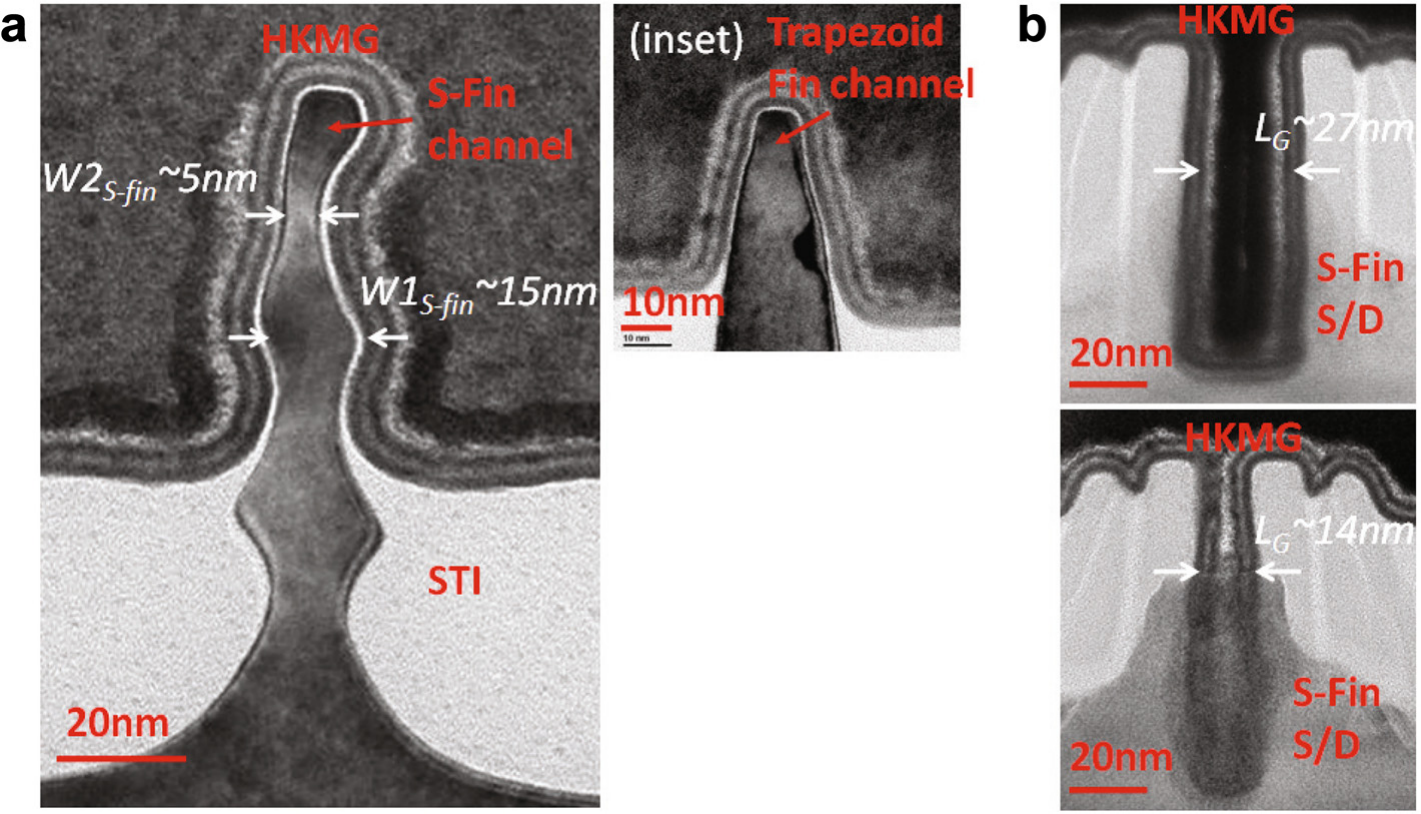
Cross-sectional TEM images of S-FinFET along different axis. **a** The cross-sectional views of fabricated S-Fin and conventional Fin in the inset with multi-layer HKMG stack. **b** The cross-sectional views of HKMG S-FinFETs with *L*_*G*_ of 27 nm and *L*_*G*_ of 14 nm

The cross-sectional views of the fabricated P-type S-FinFET along the directions of the gate and fin are shown in Fig. 3a, b, respectively. Because the fabricated N-type transistors in our lab have the gap filling issues of MGs, this letter focuses on results and discussion of P-type devices.

In Fig. 3a, the curved surface of the fin sidewall is clearly different from the straight sidewall of the conventional fins in the inset of Fig. 3a. The etched S-fin has three body and neck regions stacked from the top to the bottom of the fin, whereas two body and neck regions are above the STI oxide layer to form the fin channel of the transistor. The entire height of the S-fin channel is approximately 55 nm, deeper than the height (~40 nm) of a conventional fin for reference. *W1*_S-fin_ and *W2*_S-fin_ of the second fin body and first neck are 15 and 5 nm, respectively. *W1*_S-fin_ of the first fin body is slightly smaller owing to the process variations.

The cross-sectional views of the gate stacks created by the all-last HKMG process for the scallop-shaped fins with physical *L*_*G*_ of 27 and 14 nm are shown in Fig. 3b.

In these gate stacks, the multilayered film structure of the interface oxide/Hf-based high-k dielectric (0.7/2.3 nm) and the TiN/TaN/TiN/W (1.2/1.5/2.5/100 nm) metal gates are similar to the state-of-the-art mainstream HKMG process [2]. The film stack demonstrates good filling capability for the 27-nm gate structure. However, a filling issue for the top W metal in the 14-nm gate structure occurs, leading to a small variation in the gate’s effective work function (EWF), though both the work-function TiN layers near the gate/Fin interface are keeping good. The extracted effective oxide thickness (EOT) and EWF from measured capacitance-voltage curves on thousands of S-fins are 0.9 nm and 5.05 eV, respectively. In this figure, the interface oxide thickness is relatively uniform even on the curved surface of S-fins because the oxide is very thin (0.7 nm) and grown on them after the corner rounding process with a chemical method.

Furthermore, the gate stack in Fig. 3a demonstrated good film uniformity and conformation to the curved surface of the scallop-shaped fin. It is shown that the distribution variation of the MG EWF along the surface of the curved channel is suppressed, which is often observed in nanowire devices owing to the film-filling issues underneath the nanowire channels.

**Fig. 4:**
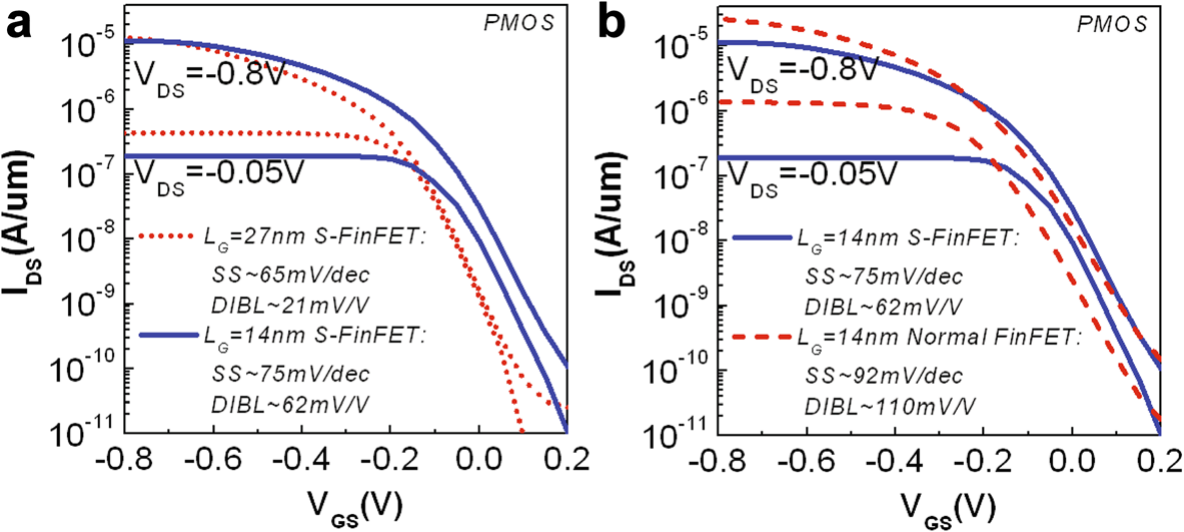
Transfer characteristics of fabricated S-FinFETs with different *L*_*G*_s and reference device. **a** *I*_DS_ *–V*_GS_ transfer curves of HKMG S-FinFETs with *L*_*G*_ of 27 nm and *L*_*G*_ of 14 nm. **b** *I*_DS_ *–V*_GS_ transfer curves of an S-FinFET and a normal FinFET with *L*_*G*_ of 14 nm and similar process

Figure 4a shows the *I*_DS_*–V*_GS_ characteristics of fabricated p-type S-FinFETs with 27-nm and 14-nm gate lengths at a supply voltage (*V*_DD_) of 0.8 V. Both saturated (*V*_DS_ = −*V*_DD_ = −0.8 V) and linear (*V*_DS_ = −0.05 V) transfer curves are shown for each device. The transfer curves are normalized by the actual channel width, which is defined as the perimeter (~118 nm/per fin) of channel cross-sectional area of two S-fins. The value of actual channel width in the S-FinFET is obviously larger than that of normal FinFET with similar fin width and height.

The *I*_DS_*–V*_GS_ curves of the reference 14 nm normal FinFET are shown in the Fig. 4b in comparison with those of S-FinFETs.

The critical electrical parameters such as drain-induced barrier lowering (DIBL) and subthreshold swing (SS) for evaluating SCE immunity of scaled transistors are defined as:

$$ \begin{array}{c}\hfill \mathrm{DIBL}=\left|{V}_{\mathrm{TH},\ \mathrm{s}\mathrm{a}\mathrm{t}}\hbox{--} {V}_{\mathrm{TH},\ \mathrm{l}\mathrm{i}\mathrm{n}}\right|/\left({V}_{\mathrm{DD}}\hbox{--}\ 0.05\mathrm{V}\right)\hfill \\ {}\hfill \mathrm{S}\mathrm{S}=\mathrm{d}\left({V}_{\mathrm{DS}}\right)/\mathrm{d}\left[ log\left({I}_{\mathrm{DS},\ \mathrm{s}\mathrm{a}\mathrm{t}}\right)\right]\hfill \end{array} $$

where *V*_TH,sat_ and *V*_TH,lin_ are threshold voltages of the device under saturated and linear conditions, respectively. Both are the mean value of devices across the wafer.

The DIBL and SS are 21 mV/V and 65 mV/dec for a 27-nm *L*_*G*_ and 62 mV/V and 75 mV/dec for a 14-nm *L*_*G*_ S-FinFET. These parameters are excellent and approximately maintained the same level as 22-nm tri-gate devices (*L*_*G*_ = 30 nm) for mass production [2], even with a smaller physical *L*_*G*_.

From Fig. 4b, it is found that *V*_TH_s of these fabricated S-FinFETs and normal FinFETs are relatively small, showing that the devices are more like depletion devices. This behavior may be due to a relative low channel doping and an extremely band-edged MG (EWF~5.05 eV) integrated on very short channels.

Although DIBL and SS are much improved, the drain current of the fabricated S-FinFET is smaller than that of reference normal FinFET in Fig. 4b. This may result from the large series resistance in S/D regions without selective epitaxy process due to the limited process capability in our lab-line. The thinner fin structure of S-FinFET has a more serious series resistance effect. As a result, it induces a smaller driving current. Another reason is perhaps due to the degraded carrier mobility for serious electrical field scattering or stress effect in the corner of the scalloped fins. The plasma damage on S-fin surface with more etch process may also induce a degradation on carrier mobility in the channel.

**Fig. 5:**
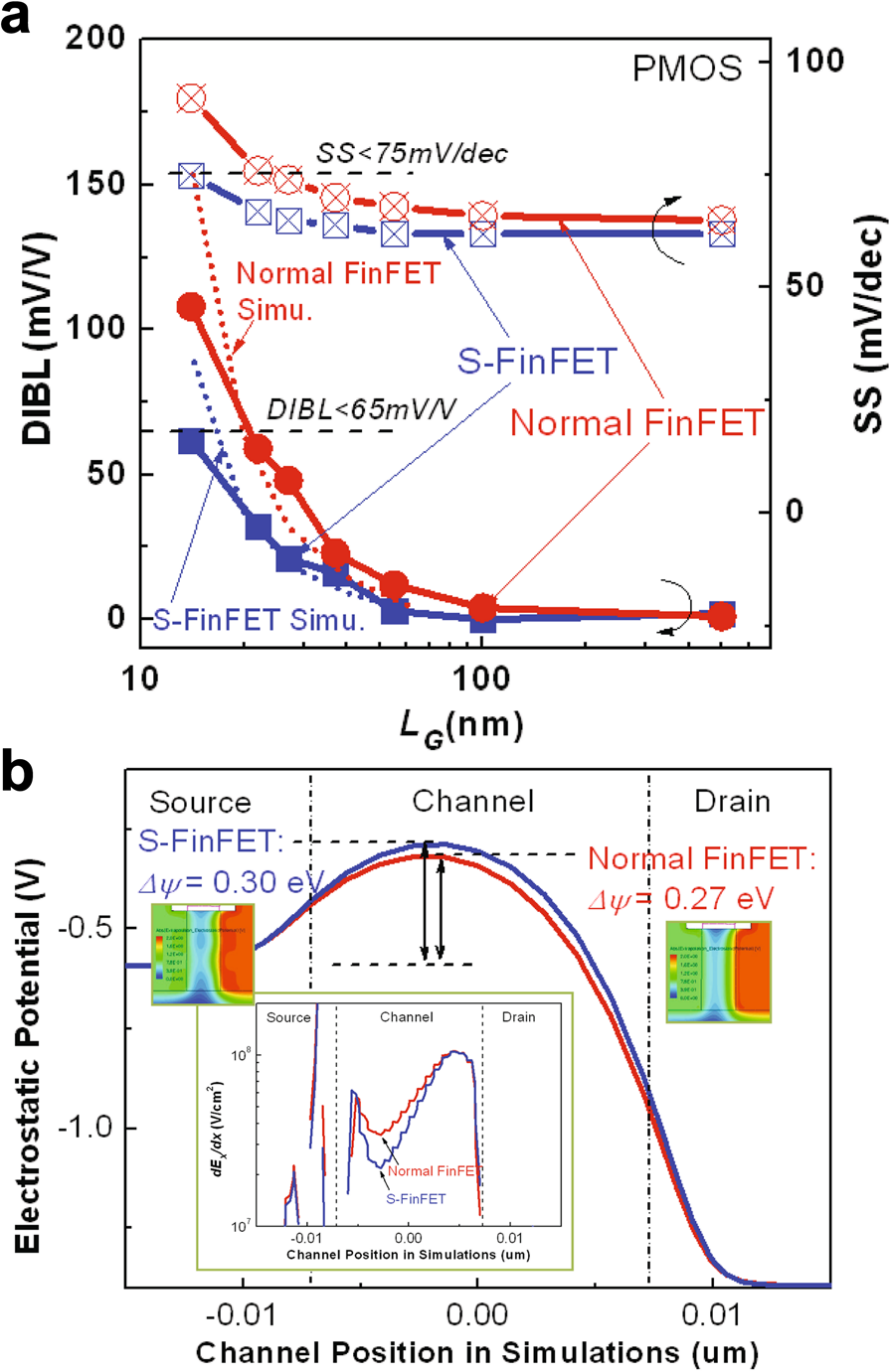
Improved SCE control parameters of DIBL and SS as the gate length scaling for fabricated S-FinFETs. **a** DIBL and SS are shown as a function of gate length for S-FinFETs and normal FinFETs. Simulated DIBL variations are included. **b** Simulated electrostatic potential as well as *dE*_*x*_ */dx* in different channels for explaining the improved DIBL in S-FinFETs

The dependencies of DIBL and SS on the *L*_*G*_ scaling for the fabricated S-FinFETs and normal FinFETs are shown in Fig. 5a.

For long channels, both S-FinFETs and normal FinFETs have a similar DIBL and SS. As the gate length scales into the sub-40-nm range, the DIBL and SS for normal FinFETs clearly increase owing to the stronger SCE, whereas there is a slight increase for the S-FinFETs. For a 27-nm *L*_*G*_, the decrease of DIBL and SS for S-FinFETs over normal FinFETs are 27 mV/V and 9 mV/dec, respectively. For a 14-nm *L*_*G*_, the decrease of DIBL and SS are 48 mV/V and 17 mV/dec, respectively.

Because the S-FinFET and normal FinFET have similar channel PTS doping (peak concentration of 3 × 10^18^ cm^−3^ located below STI surface), S/D doping, activation process (1050 °C Spike RTA), and MG EWF, the improvements of DIBL and SS are mainly attributed to the channel structure modification via the introduction of an enhanced gate electric field in the special fin channel. As shown in Fig. 5a, the DIBL and SS of the S-FinFETs are slightly degraded but still do not exceed the minimum acceptable criteria for low-power CMOS circuit applications as *L*_*G*_ continuously scales down 14 nm, which is the critical specifications of the sub-10-nm CMOS process node. However, the parameters of the normal FinFET are greatly degraded, much worse than those of the S-FinFET, far away from the acceptable level for CMOS circuit applications. The simulated DIBL variations with *L*_*G*_ for these two devices are also shown in Fig. 5a. The trends are very close to these experimental results, which confirmed the structure advantage of S-FinFET.

To insightfully illustrate the physical reason of improved DIBL in S-FinFET, the simulated electrostatic potential profiles in the channels by TCAD are shown in Fig. 5b. In this figure, with the same drain voltage of 0.8 V, the energy barrier (0.30 eV) in the source for S-FinFET is larger than that (0.27 eV) for normal FinFET. Meanwhile, in the inset of Fig. 5b, the gradient of lateral electrical field (*dE*_*x*_*/dx*) in S-FinFET channel is obviously smaller than that in normal FinFET channel. These parameter differences decide the parasitic charges controlled by drain electrical field in the channel of S-FinFET fewer than those of normal FinFET, which causes the device to have a smaller DIBL value [11].

**Fig. 6:**
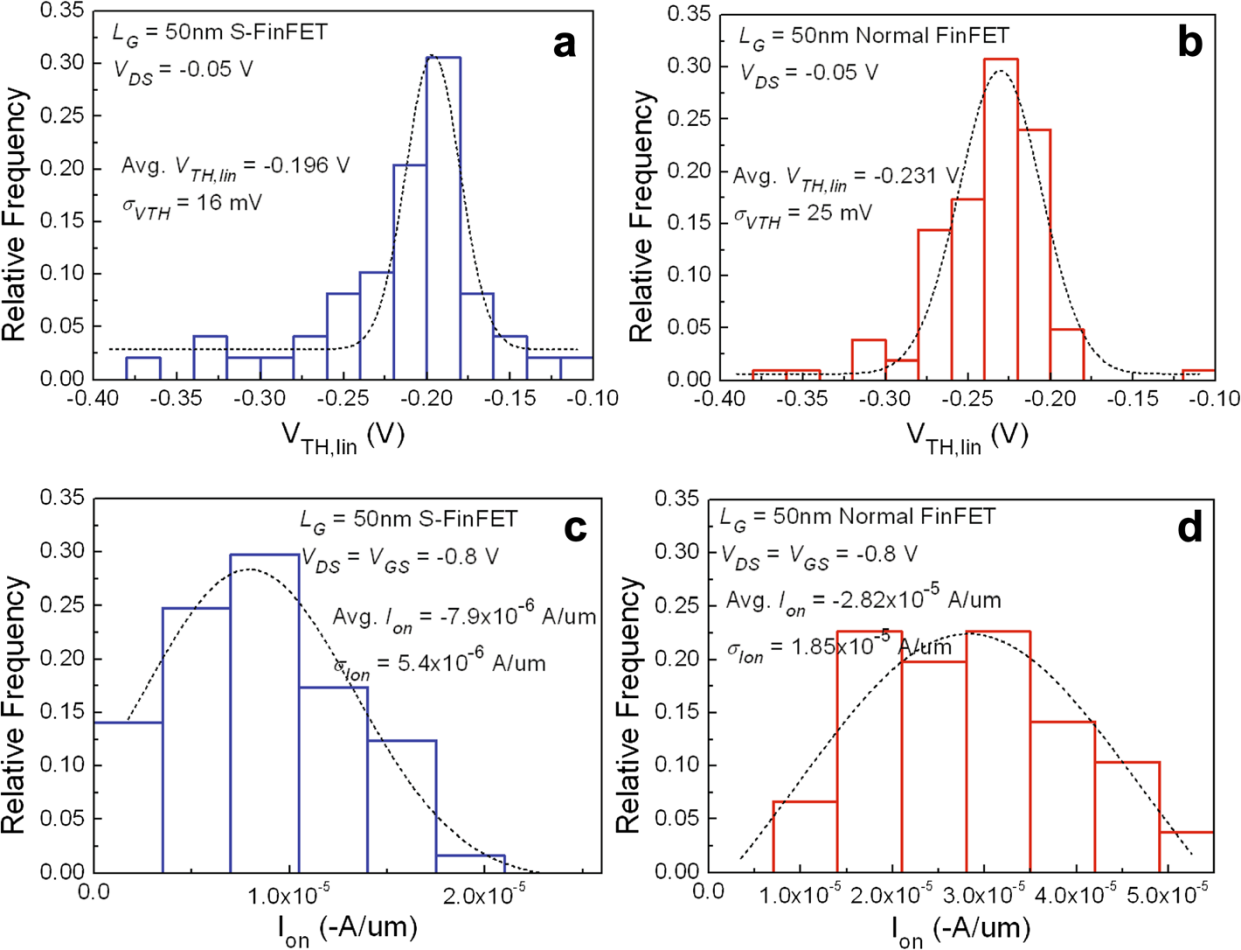
Variability of *V*_TH,lin_ and *I*_on_ for fabricated 50 nm S-FinFETs and normal FinFETs. The measured variability of *V*_TH,lin_ between **a** S-FinFETs and **b** normal FinFETs. The measured variability of *I*_on_ between **c** S-FinFETs and **d** normal FinFETs

The variability of *V*_TH,lin_ and on-state current (*I*_on_) for 50 nm S-FinFETs and normal FinFETs are shown in Fig. 6. From these figures, the variation σ of fabricated S-FinFETs is similar to those of normal FinFETs. The reason maybe the variability of FinFETs are mainly determined by four sources [12]: variation of (a) gate length, (b) fin thickness or width, (c) channel doping, and (d) EWF of MG. The variations of (b) in S-FinFETs are increased for scalloped channel shape, but those of (c) are decreased due to attenuated heavily doping region near fin bottoms. In addition, the most important factor of the variation in EWF of MGs is the same for both devices. As a result, the variability in fabricated S-FinFETs demonstrates no obvious degradations.

These results indicate that the S-FinFET demonstrates an obvious scaling advantage over the normal FinFET for sub-10-nm node CMOS process applications.

## Conclusions

Novel S-FinFETs with mainstream all-last HKMG technology using special fin channels have been reported. By slightly modifying the fin etching process of the normal FinFET process, the new devices have achieved excellent DIBL and SS as *L*_*G*_ scaled down below 20 nm. The variability has no obvious degradation. Our results are promising for helping general FinFET technology extend into the sub-10-nm node.
